# NNT-AS1 modulates prostate cancer cell proliferation, apoptosis and migration through miR-496/DDIT4 axis

**DOI:** 10.1186/s12935-020-01505-3

**Published:** 2020-09-24

**Authors:** Changlei Yao, Xianghua Cheng, Xiuquan Guo, Xulou Lu, Fan Bu, Yanfen Xu

**Affiliations:** 1 Department of Urinary Surgery, People’s Hospital of Rizhao, No.126, Tai an Street, Dong Gang District, Rizhao, 276826 Shandong China; 2 Department of Surgery 2, People’s Hospital of Rizhao, No.126, Tai an Street, Dong Gang District, Rizhao, 276826 Shandong China

**Keywords:** Prostate cancer, NNT-AS1, miR-496, DDIT4

## Abstract

**Background:**

Emerging studies have disclosed long non-coding RNAs (lncRNAs) as pivotal modulators in the progression of prostate cancer (PCa). Current research planned to figure out the involvement of lncRNA nicotinamide nucleotide transhydrogenase antisense RNA 1 (NNT-AS1) in PCa.

**Methods:**

RNA expression was examined using RT-qPCR in PCa cells. Functional assays assessed the viability, proliferation, apoptosis and migration of PCa cells. RNA pull down and luciferase reporter experiments detected the interplay between miRNA and lncRNA or mRNA.

**Results:**

NNT-AS1 was apparently upregulated in PCa cells. NNT-AS1 deficiency abrogated PCa cell viability, proliferation and migration but promoted apoptosis. Besides, miR-496 could be sequestered by NNT-AS1 to elevate the expression of DNA damage inducible transcript 4 (DDIT4) in PCa. Rescue assays indicated that overexpressed DDIT4 or restrained miR-496 could reverse the influence of NNT-AS1 depletion on malignant processes in PCa cells.

**Conclusion:**

NNT-AS1 contributes to the malignant phenotypes of PCa cells through targeting miR-496 to boost DDIT4 expression.

## Background

Prostate cancer (PCa) is a frequent malignancy with high mortality amongst men all over the world [1, 2]. The overall survival rate of PCa patients in USA is on the rise, but the incidence of PCa has not declined globally, particularly in East Asia [3, 4]. Therefore, the exploration of effective molecular targets for PCa may contribute to understand the pathogenesis of PCa, eventually helping to develop new strategies for PCa treatment.

Long non-coding RNAs (lncRNAs) are a group of transcripts with the length over 200 nt and without the capacity to encode protein [5, 6]. In human cancers, lncRNAs play crucial parts in biological processes including cell differentiation, proliferation, apoptosis and migration. For instance, lncRNA HOTTIP facilitates cell proliferation and migration in pancreatic cancer [7]. CUDR facilitates liver cancer stem cell growth via its regulation on the expression of TERT and C-Myc [8]. LncRNA SPRY4-IT1 aggravates the development of human melanoma [9]. It is also reported that lncRNA NNT-AS1 with abnormal regulation is implicated in diverse cancers, such as osteosarcoma [10], non-small cell lung cancer [11] and colorectal cancer [12]. In our study, we wondered NNT-AS1 expression and its effect in PCa.

Besides, increasing evidence has indicated that microRNAs (miRNAs) are considerable players in cancer biology. MiR-20a enhances cell proliferation and invasion by regulating APP in human ovarian cancer [13]. MiR-4458 inhibits the progression of human lung cancer through targeting Lin28B [14]. In present study, miR-496 was predicted to combine with NNT-AS1, and DDIT4 acted as the direct gene of miR-496. Thus we further explored the possible mechanism involving NNT-AS1, miR-496 and DDIT4 underlying PCa progression.

In short, our study intended to explore the function and probable molecular mechanism of NNT-AS1 in PCa, which might contribute to therapy development for prostate cancer.

## Methods

### Cell lines

Human prostate cancer cell lines (LNCaP clone FGC, VCaP, LNCaP C4-2B, PC3) and human normal prostate epithelial cell line (RWPE-1) were procured from ATCC (Manassas, VA, USA). They were routinely grown in DMEM (Gibco, Rockville, MD, USA) with 1% antibiotics (Gibco) and 10% FBS (Gibco), under 5% CO_2_ at 37 ℃.

### Real-time quantitative polymerase chain reaction (RT-qPCR)

The total RNA was separated from cells by use of TRIzol Reagent (Invitrogen, Carlsbad CA), and then converted to cDNA using PrimeScript Reverse Transcriptase Kit (Takara, Shiga, Japan). RT-qPCR analysis was undertaken with PrimeScript Reverse Transcriptase Kit (Takara). Gene expression was processed with 2^−ΔΔCt^ method after standardized to U6 snRNA or GAPDH mRNA. This experiment was repeated at least three times.

### Cell transfection

The shRNAs designed to target NNT-AS1 (sh-NNT-AS1#1/2) or DDIT4 (sh-DDIT4#1/2) and the relevant nonspecific shRNAs (sh-NC), as well as pcDNA3.1-DDIT4 and empty vector (pcDNA3.1-NC), all were procured from Genechem (Shanghai, China). The miR-496 mimics/inhibitor and NC mimics/inhibitor were constructed by GenePharma (Shanghai, China). VCaP and PC3 cells were transfected with indicated plasmids for 48 h in the presence of Lipofectamine 3000 (Invitrogen).

### Cell counting kit-8 (CCK-8) assay

The survival curve of PCa cells under diverse conditions were examined by CCK-8 assay as per the user’s guide. In short, 1 × 10^3^ cells were seeded into a 96-well plate and then incubated for indicated times (24, 48, 72 h). After that, cells were processed with 10 μL of CCK-8 reagent for another 4 h. Absorbance at 450 nm was assessed via a microplate reader (Olympus, Tokyo, Japan). The experiment was repeated at least three times.

### 5-Ethynyl-2′-deoxyuridine (EdU) assay

EdU assay was undertaken in VCaP and PC3 cells by employing the BeyoClick™ EdU Cell Proliferation Kit (Beyotime, Shanghai, China) with Alexa Fluor 594. After transfection, cells were collected and incubated in EdU medium for 2 h, followed by treatment with DAPI staining. After washing, the stained cells were captured via inverted microscope (Olympus, Tokyo, Japan). This experiment was repeated at least three times.

### Colony formation assay

After 48 h of transfection, VCaP and PC3 cells were placed in 6-well plates for 14 days. Then, colonies were fixated by 4% PFA for 30 min and colored by crystal violet solution for 5 min. The visible clones were imaged and counted manually. This experiment was repeated at least three times.

### Flow cytometry analysis

The apoptosis of VCaP and PC3 cells was assessed using Annexin V/PI double staining kit (Invitrogen). After transfection, cells were reaped and re-suspended in Binding Buffer, and then double-stained in the dark for 15 min. After washing in PBS, the apoptotic cells were analyzed by flow cytometry (BD Biosciences, Franklin Lakes, NJ). The experiment was repeated at least three times.

### JC-1 assay

The mitochondrial membrane potential (Δψm) was analyzed via JC-1 assay, in line with the instruction of JC-1 dye (Beyotime, Shanghai, China). Transfected cells were placed in 12-well plates for 24 h, and then treated with 2.5 µg/mL of JC-1 for 30 min at 37 ℃. After washing plates by PBS, the microscope (Olympus) was used for the observation and analysis of dyed cells. The experiment was repeated at least three times.

### Wound healing assay

The transfected PCa cells were cultured in 6-well plates until nearly 90% confluence, followed by the creation of wounds using 200 μL of pipette tip. After culturing in serum-free medium for 0 and 24 h, the wound gaps were assessed, and wound closure images were acquired under a microscope (Olympus). The experiment was repeated at least three times.

### Subcellular fractionation

The NNT-AS1 content in cell cytoplasm and cell nucleus of VCaP and PC3 cells was studied by subcellular fractionation using PARIS™ Kit (Invitrogen). After cells treated with cell fractionation buffer and then centrifuged, qRT-PCR was applied to determine the expression levels of NNT-AS1 in cytoplasmic and nuclear fractions, with GAPDH and U6 as respective controls. The experiment was repeated at least three times.

### FISH

The subcellular distribution of NNT-AS1, miR-496 and DDIT4 mRNA in VCaP and PC3 cells was estimated by FISH assay using specific RNA FISH probes (Ribobio, Guangzhou, China). After fixing and digesting, the air-dried cell samples were incubated with indicated probes diluted in the hybridization buffer. Finally, cells were imaged and analyzed using microscope (Olympus). This experiment was repeated at least three times.

### MS2-RIP assay

For MS2-RIP assay, cell samples were transfected with pcDNA3.1-MS2-NNT-AS1 or pcDNA3.1-MS2 for 48 h. Then, samples were used for RIP assay with anti-GFP antibody (Abcam, Cambridge, MA), using Magna RIP RNA-Binding Protein Immunoprecipitation Kit (Millipore, Bedford, MA). The precipitated RNAs were finally monitored by RT-qPCR. The experiment was repeated at least three times.

### RNA pull down assay

The interaction between RNAs was also estimated by RNA pull down assay by use of Pierce Magnetic RNA-Protein Pull-Down Kit (Thermo Fisher Scientific, Waltham, MA). After mixing the cell lysates with biotinylated RNA probes and magnetic beads, RNAs in the pulled-down mixture were collected for qRT-PCR analysis. The experiment was repeated at least three times.

### Luciferase reporter assay

Sequences of full-length NNT-AS1 or DDIT4 3′UTR covering the wild-type and mutant miR-496 binding sites were enclosed into the pmirGLO luciferase vectors (Promega, Madison, WI, USA). The acquired constructs were co-transfected into VCaP and PC3 cells with miR-496 mimics or NC mimics for 48 h, and then the luciferase activity of indicated groups was studied using luciferase reporter assay system (Promega). The experiment was repeated at least three times.

### Statistical analyses

Each experiment of our research was repeated at least three times. Data from all the individually conducted experiments were given as the mean ± SD. Difference between groups was compared by Student’s t-test or one-way ANOVA, by employing GraphPad Prism 7 (GraphPad Software, Inc., La Jolla, CA). The data were regarded as significant when p < 0.05.

## Results

### NNT-AS1 knockdown inhibits PCa cell proliferation and migration

**Fig. 1:**
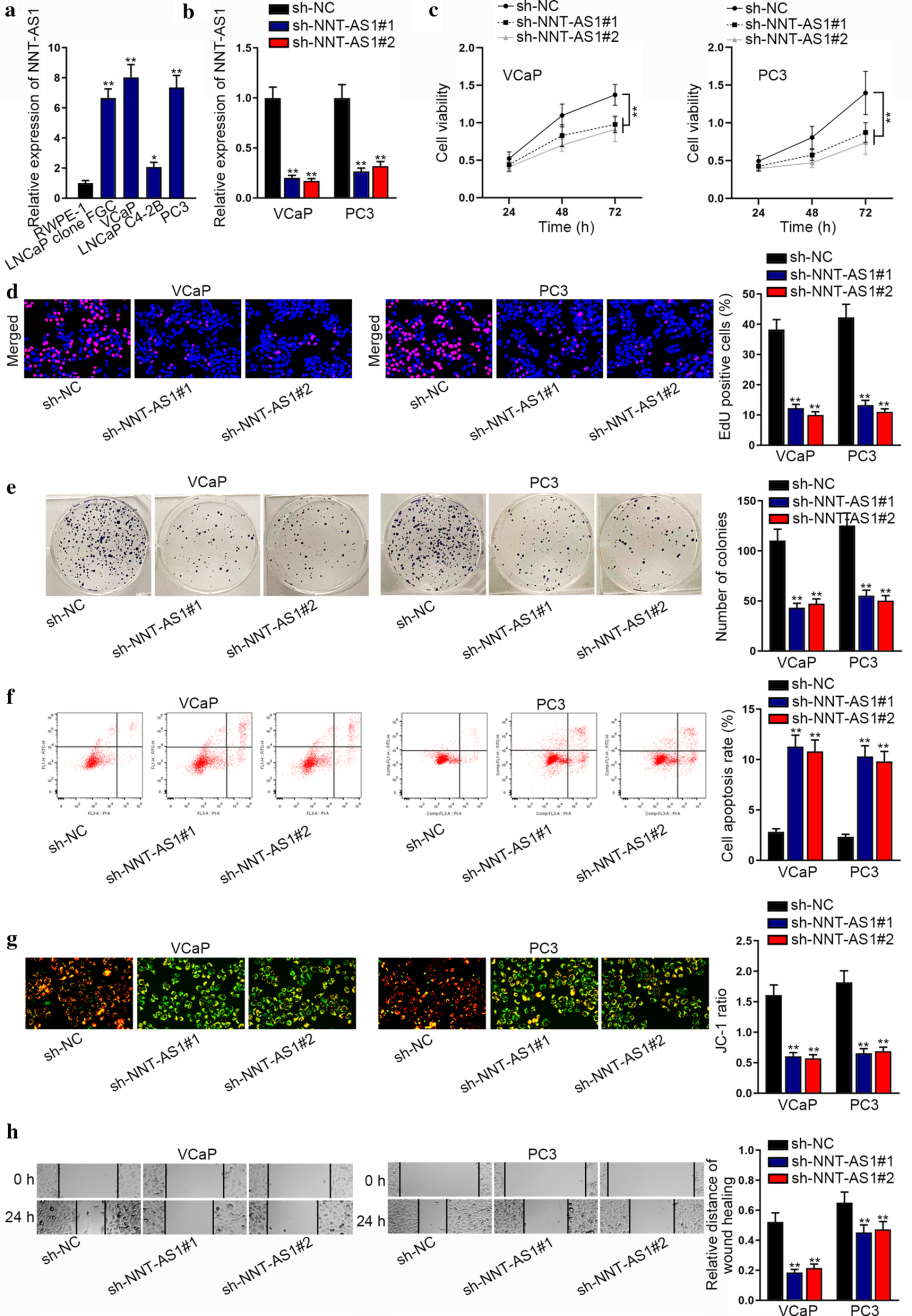
NNT-AS1 knockdown inhibited PCa cell viability, proliferation, migration and promoted apoptosis. **a** NNT-AS1 expression was examined using RT-qPCR in PCa cells. **b** NNT-AS1 expression was detected by RT-qPCR in PCa cells transfected with shRNAs targeting NNT-AS1. **c** CCK-8 assay indicated cell viability when NNT-AS1 was down-regulated in VCaP and PC3 cells. **d**, **e** EdU and colony formation assays measured cell proliferation in VCaP and PC3 cells with or without NNT-AS1 inhibition. **f**, **g** Flow cytometry analysis and JC-1 assay examined the apoptosis rate of VCaP and PC3 cells with or without NNT-AS1 silence. **h** Wound healing assay displayed the migration capacity of VCaP and PC3 cells with silenced NNT-AS1 or not. *p < 0.05, **p < 0.01

To identify the role of NNT-AS1 in PCa, we measured NNT-AS1 expression in PCa cells for the first step. As shown in Fig. 1a, NNT-AS1 was overexpressed in LNCaP clone FGC, VCaP, LNCaP C4-2B and PC3 cells relative to the normal RWPE-1 cells. Considering relatively higher NNT-AS1 level in VCaP and PC3 cells, we selected these two kinds of PCa cells for follow-up experiments. Thereafter, we employed shRNAs to interfere NNT-AS1 expression in VCaP and PC3 cells, and RT-qPCR results evidenced the successful silence of NNT-AS1 in these two cells (Fig. 1b). Then we designed and conducted a series of loss-of-function assays. As a result, we found that loss of NNT-AS1 led to abrogated viability of VCaP and PC3 cells (Fig. 1c). Besides, the results of EdU assay indicated that NNT-AS1 suppression declined the proportion of VCaP and PC3 cells with positive EdU (Fig. 1d). Additionally, the results of colony formation assay further proved the suppression of NNT-AS1 depletion on PCa cell proliferation (Fig. 1e). Next, flow cytometry analysis demonstrated that silencing NNT-AS1 accelerated the apoptosis of both VCaP and PC3 cells (Fig. 1f). Further, such phenomenon was also testified by the results of JC-1 assay (Fig. 1g). Moreover, it was displayed that NNT-AS1 depletion decreased the migratory capacity of VCaP and PC3 cells (Fig. 1h). Together, NNT-AS1 silence hinders PCa cell proliferation and migration.

### NNT-AS1 sponged miR-496 in PCa cells

**Fig. 2:**
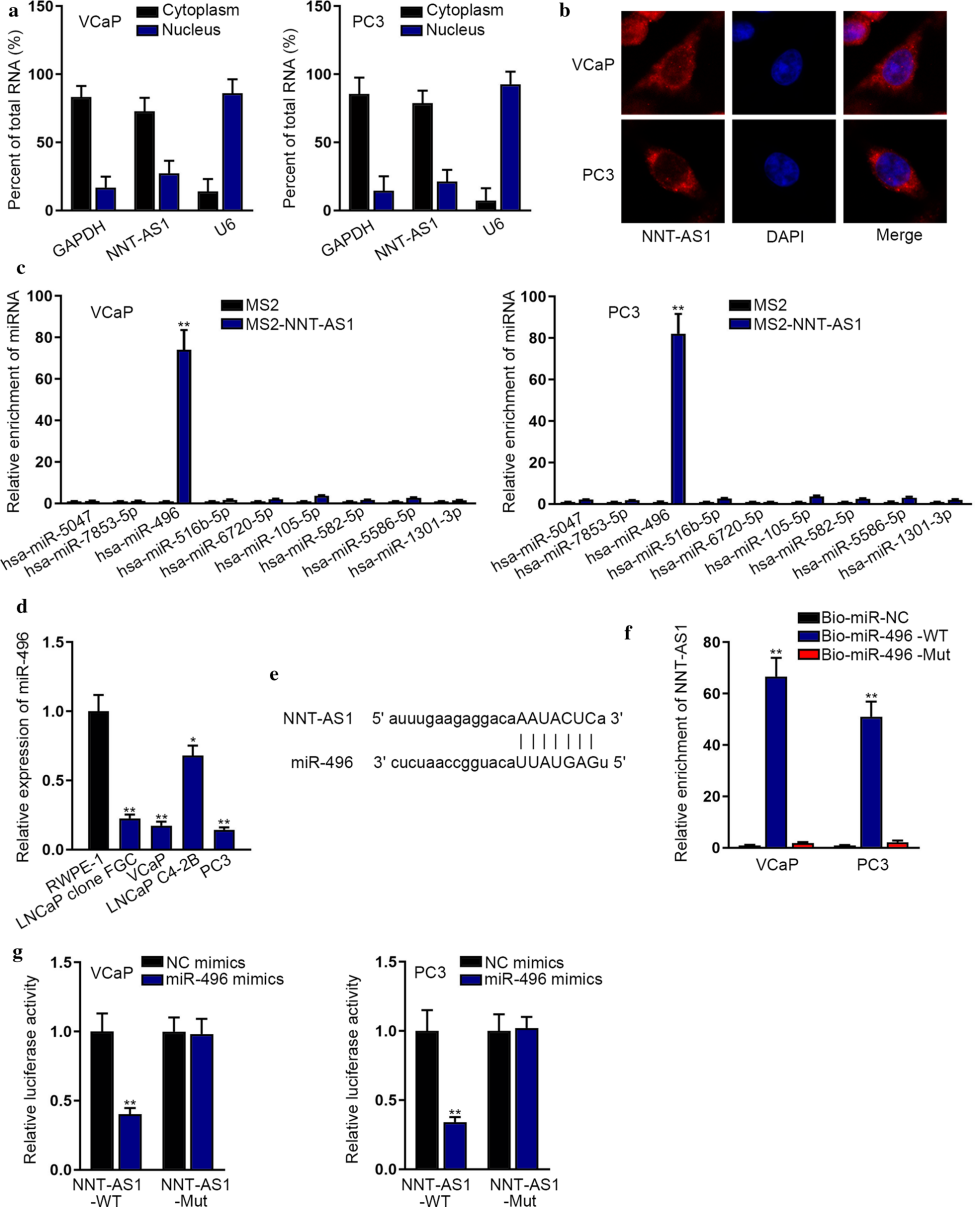
NNT-AS1 served as miR-496 sponge in PCa cells. **a** Subcellular fractionation assay demonstrated the location of NNT-AS1 in PCa cells. **b** FISH experiments further proved the distribution of NNT-AS1 in PCa cells. **c** MS2-RIP assays were conducted in both VCaP and PC3 cells to assess the binding of 9 miRNA candidates toNNT-AS1. **d** RT-qPCR detected the expression of miR-496 in PCa cells. **e** ENCORI revealed the binding sites between NNT-AS1 and miR-496. **f** RNA pull down assay was performed to attest the enrichment of NNT-AS1 in indicated groups. **g** Luciferase reporter assay further validated the interaction between NNT-AS1 and miR-496 in VCaP and PC3 cells. *p < 0.05, **p < 0.01

To determine the subcellular location of NNT-AS1 in PCa cells, we applied subcellular fractionation and FISH experiments. Results displayed that NNT-AS1 was primarily existed in the cytoplasm (Fig. 2a). At the same time, FISH assay also proved the main distribution of NNT-AS1 in the cytoplasmic fraction of PCa cells (Fig. 2b). Previous studies have indicated that lncRNAs can serve as a competing endogenous RNA (ceRNA) via sponging miRNA to post-transcriptionally release mRNAs targeted by this miRNA [15]. Accordingly, we speculated that NNT-AS1 might act as a ceRNA in PCa through sponging certain miRNA. In this regard, we utilized ENCORI (https://starbase.sysu.edu.cn) to search for probable miRNAs and screened out nine miRNAs that could bind with NNT-AS1. To test the binding affinity of NNT-AS1 with above miRNAs, MS2-RIP assays were conducted in both VCaP and PC3 cells. Interestingly, results disclosed the highest enrichment of miR-496 in MS2-NNT-AS1 groups (Fig. 2c). Further, we also validated the existence of miR-496 in the compounds precipitated by MS2-NNT-AS1 via agarose gel electrophoresis (Additional file 1: Figure S1A). Moreover, we discovered that miR-496 predominantly distributed in the cytoplasm of VCaP and PC3 cells (Additional file 1: Figure S1B), which further indicated miR-496 as the downstream molecule of NNT-AS1 in PCa. Next, opposite to NNT-AS1, we found that the expression of miR-496 was obviously declined in PCa cells compared to that in RWPE-1 cells (Additional file 1: Fig. 2d). Then, ENCORI (https://starbase.sysu.edu.cn) revealed the binding sites between NNT-AS1 and miR-496 (Fig. 2e). Of note, the results of RNA pull down assay attested that NNT-AS1 was significantly enriched in Bio-miR-496-WT groups (Fig. 2f). Subsequently, we further validated that miR-496 overexpression remarkably abated the luciferase activity of NNT-AS1-WT reporter, but had no inhibitory effects on that of the mutated groups (Fig. 2g). All these results showed that NNT-AS1 serves as the sponge of miR-496 in PCa.

### DDIT4 is the target downstream of miR-496 in PCa cells

**Fig. 3:**
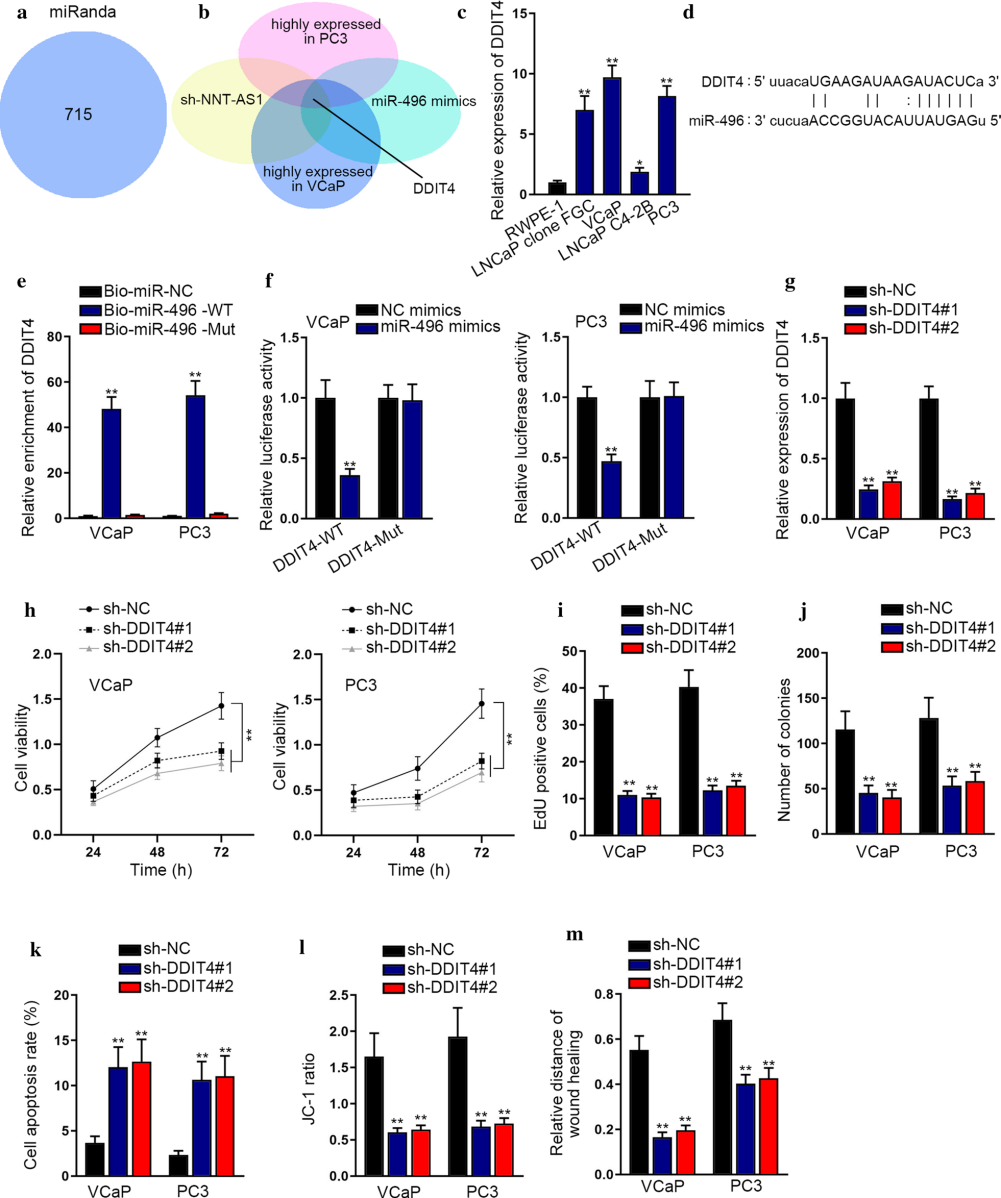
DDIT4 was the direct target of miR-496 in PCa cells. **a** MiRanda found out seven hundred and fifteen mRNAs that might bind with miR-496. **b** RT-qPCR selected mRNAs whose expression conformed to following four conditions: highly expressed in PC3 cells, inhibited by miR-496 mimics, highly expressed in VCaP cells and suppressed by sh-NNT-AS1. **c** RT-qPCR examined the expression of DDIT4 in PCa cells. **d** ENCORI predicted the binding sites between DDIT4 and miR-496. **e** RNA pull down experiments demonstrated that the abundance of DDIT4 in indicated groups. **f** Luciferase reporter assays examined the relative luciferase activity in VCaP and PC3 cells with different transfections. **g** The expression of DDIT4 was detected via RT-qPCR in VCaP and PC3 cells transfected with shRNAs targeting DDIT4. H. CCK-8 assay indicated cell viability when DDIT4 was silenced in VCaP and PC3 cells. **i**, **j** EdU and colony formation assays detected the proliferation of VCaP and PC3 cells with DDIT4 inhibition. **k**, **l** Flow cytometry analysis and JC-1 assay evaluated the apoptosis of VCaP and PC3 cells with DDIT4 suppression. **m** Wound healing assay displayed the migration of VCaP and PC3 cells under DDIT4 down-regulation. *p < 0.05, **p < 0.01

To explore the downstream molecule targeted by miR-496, we analyzed miRanda and found out seven hundred and fifteen messenger RNAs (mRNAs) that might be recognized by miR-496 (Fig. 3a). Then RT-qPCR was employed to select mRNAs whose expression conformed to following four conditions: highly expressed in PC3 cells, downregulated by miR-496 mimics, highly expressed in VCaP cells and suppressed by sh-NNT-AS1. As a consequence, DDIT4 was screened out as the suitable downstream target of miR-496 in PCa (Fig. 3b). Besides, we unveiled that DDIT4 level was apparently boosted in PCa cells relative to RWPE-1 cells (Fig. 3c). Also, it was revealed that DDIT4 mRNA largely occupied in the cytoplasm of VCaP and PC3 cells (Additional file 1: Figure S1C). Based on these results, we recognized DDIT4 mRNA as the target of miR-496 in PCa. Subsequently, we predicted the binding sites between DDIT4 and miR-496 using ENCORI (Fig. 3d). To evaluate the relationship between DDIT4 and miR-496, we further conducted RNA pull down experiments. As expected, we observed abundant enrichment of DDIT4 in Bio-miR-496-WT groups (Fig. 3e). Then luciferase reporter assays unveiled that the relative luciferase activity was lowered in VCaP and PC3 cells co-transfected with miR-496 mimics and DDIT4-WT, while no apparent alteration of that was discovered in VCaP and PC3 cells co-transfected with miR-496 mimics and DDIT4-Mut (Fig. 3f). To find the part of DDIT4 played in PCa, we first knocked down its expression in VCaP and PC3 cells (Fig. 3g). Then CCK8 assay indicated that DDIT4 silencing hindered the viability of both VCaP and PC3 cells (Fig. 3h). Following, EdU and colony formation assays suggested the abated cell proliferation in DDIT4-silenced groups (Fig. 3i, j). After that, the results of flow cytometry analysis and JC-1 assay revealed that suppressing DDIT4 elevated the apoptosis rate of VCaP and PC3 cells (Fig. 3k, l). Moreover, wound healing assay displayed that down-regulation of DDIT4 hindered the migration of VCaP and PC3 cells (Fig. 3m). Above data manifested that DDIT4 is the factor directly downstream of miR-496 and exerts a tumor-inhibitory function in PCa.

### NNT-AS1 accelerates PCa cell proliferation and migration via targeting miR-496/DDIT4 signaling

**Fig. 4:**
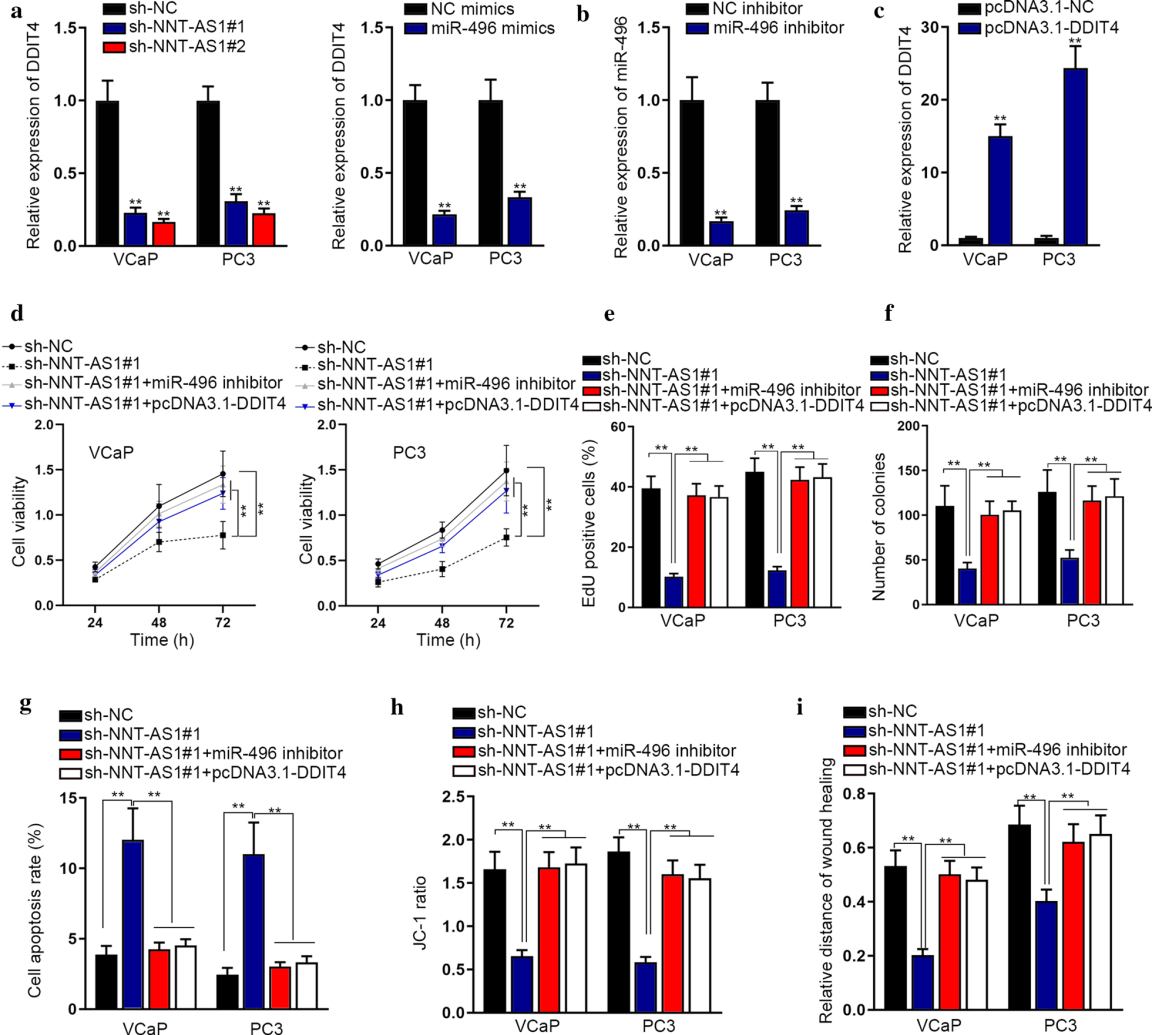
NNT-AS1 promoted the malignant behaviors in PCa cells through targeting miR-496/DDIT4 pathway. **a** The expression of DDIT4 was detected using RT-qPCR in VCaP and PC3 cells under NNT-AS1 silence or miR-496 enhancement. **b**, **c** RT-qPCR examined miR-496 expression in PCa cells after transfection with miR-496 inhibitor or NC inhibitor, and DDIT4 expression in cells with transfection of pcDNA3.1-DDIT4 or pcDNA3.1-NC. **d** CCK-8 assay examined the viability of VCaP and PC3 cells transfected with sh-NC, sh-NNT-AS1#1, sh-NNT-AS1#1 + miR-496 inhibitor, or sh-NNT-AS1#1 + pcDNA3.1-DDIT4. **e**, **f** EdU and colony formation assays measured the proliferation of VCaP and PC3 cells under above conditions. **g** Flow cytometry assay assessed the apoptosis rate in indicated VCaP and PC3 cells. **h** JC-1 assay detected cell apoptosis in indicated VCaP and PC3 cells. **i** Wound healing experiments demonstrated the migration ability of indicated VCaP and PC3 cells. **p < 0.01

To identify whether NNT-AS1 worked in PCa via modulating miR-496 and DDIT4, rescue experiments were conducted. We first detected that the expression of DDIT4 was evidently lessened by NNT-AS1 inhibition or miR-496 promotion in VCaP and PC3 cells (Fig. 4a). Besides, miR-496 expression was markedly reduced by miR-496 inhibitor (Fig. 4b), while DDIT4 expression was obviously boosted by pcDNA3.1-DDIT4 in VCaP and PC3 cells (Fig. 4c). Then it was revealed that the inhibited viability and proliferation of NNT-AS1-silenced VCaP and PC3 cells were completely reversed by miR-496 inhibition or DDIT4 enhancement (Fig. 4d–f). Meanwhile, we also unveiled that the elevated apoptosis rate of VCaP and PC3 cells responding to NNT-AS1 knockdown was recovered under miR-496 suppression or DDIT4 up-regulation (Fig. 4g, h). Moreover, the migration ability impeded by NNT-AS1 interference was also rescued by down-regulated miR-496 or upregulated DDIT4 (Fig. 4i). In sum, NNT-AS1 aggravates the malignant phenotypes of PCa cells through sequestering miR-496 to augment DDIT4 expression.

## Discussion

As a malignancy frequently diagnosed in men, PCa has a high mortality worldwide. A previous research proposed that better understanding of characteristics in different phases during the natural history of PCa is helpful for optimal therapeutic options [16]. Further, a novel therapeutic strategy based on gene interference has been highlighted as a new potential option for treating PCa [17]. As a class of ncRNAs, miRNAs which have been reported to have pivotal functions in PCa [18], are regarded as therapeutic tool in PCa [17]. In recent years, lncRNAs, another kind of ncRNAs, have been emerged as cancer suppressors or promoters in PCa. For instance, lncRNA UCA1 facilitates PCa progression by absorbing miR-204 to regulate ATF2 [19]. LncRNA TINCR acts as a tumor suppressor and has a vital clinical value in PCa [20]. LncRNA NNT-AS1, the topic of this research, has already been uncovered to take part in diverse cancers. For example, NNT-AS1 contributes to the tumorigenesis and progression of gastric cancer through targeting miR-424/E2F1 [21]. LncRNA NNT-AS1 up-regulates CDK6 to promote progression in hepatocellular carcinoma by targeting miR-363 [22]. In our work, we discovered the effectively up-regulated NNT-AS1 in PCa cells, and knockdown of NNT-AS1 hindered cell viability, proliferation and migration while induced cell apoptosis in PCa.

In addition, lncRNAs have been thought to be potentially implicated in cancer development through binding with miRNAs. LOC134466/hsa-miR-196a-5p/TAC1 axis promotes cell proliferation of endometrial carcinoma [23]. Linc00472 suppresses tumor growth through sponging miR-196a to elevate PDCD4 expression in colorectal cancer [24]. LncRNA DANCR facilitates lung adenocarcinoma progression by regulating miR-496/mTOR pathway [25]. Consistent with these reports, our study also recognized miR-496 as the downstream factor of NNT-AS1 in PCa. Intriguingly, miR-496 has been unveiled to have different functions in different cancer types. It restrains tumorigenesis in non-small cell lung cancer [26], osteosarcoma [27] and acute myeloid leukemia [28], but serves as an oncomiR in colorectal cancer [29] and glioma [30]. Presently, we found miR-496 was downregulated in PCa cell lines, consistent with the finding of a latest study [31]. Besides, a work accomplished by Li et al. also indicates the suppressive impact of miR-496 on PCa development [32]. Similarly, our study proved that inhibiting miR-496 rescued the repressed malignancy in NNT-AS1-silenced PCa cells.

Moreover, we also explored the downstream target of miR-496 in PCa. Previously, DDIT4 has been registered to exert a pivotal part in the proliferation and tumorigenesis of gastric cancer [33]. More importantly, DDIT4 is described as an independent indicator for a poor prognosis in several malignancies [34], such as acute myeloid leukemia, breast cancer, glioblastoma multiforme, colon, skin and lung cancer. All these evidences suggest DDIT4 as a tumor promoter. In current study, we disclosed that DDIT4 was directly targeted by miR-496. Moreover, silenced DDIT4 apparently reduced cell viability, proliferation and migration in PCa, and its overexpression recovered the influence of silenced NNT-AS1 on the behaviors of PCa cells. These data suggested that NNT-AS1/miR-496/DDIT4 regulatory axis play a tumor-contributing part in PCa.

## Conclusion

In conclusion, our study elucidated the contributing role of NNT-AS1 in PCa via miR-496/DDIT4 axis, which might help for developing a novel therapeutic target for PCa.

## Supplementary information

**Additional file 1** A. The blots of agarose gel electrophoresis for the evaluation of indicated miRNAs in the complexes from MS2-NNT-AS1 groups in MS2-RIP assays. B, C. FISH analyzed the cellular location of miR-496 and DDIT4 mRNA in VCaP and PC3 cells.

## Data Availability

Relevant data and materials have been presented within the manuscript and additional files.

## Abbreviations

lncRNAs: Long non-coding RNAs
mRNA: Messenger RNA
ceRNAs: Competing endogenous RNAs
miRNA: MicroRNA
NNT-AS1: Nicotinamide nucleotide transhydrogenase antisense RNA 1
DDIT4: DNA damage inducible transcript 4
PCa: Prostate cancer
ATCC: American Type Culture Collection
DMEM: Dulbecco’s modification of Eagle’s medium
RT-qPCR: Real-time quantitative polymerase chain reaction
FBS: Fatal Bovine Serum
EdU: 5-Ethynyl-2′-deoxyuridine
DAPI: 4′,6-diamidino-2-phenylindole
FISH: Fluorescence in situ hybridization
RIP: RNA immunoprecipitation
PFA: Paraformaldehyde
Wt: Wild-type
Mut: Mutant
SD: Standard deviation
ANOVA: Analysis of Variance
