# Supplementary figures and images for: NNT-AS1 modulates prostate cancer cell proliferation, apoptosis and migration through miR-496/DDIT4 axis
Source: Cancer Cell Int. 2020 Sep 24;20:463. doi: 10.1186/s12935-020-01505-3 (PMC7513494; doi:10.1186/s12935-020-01505-3)

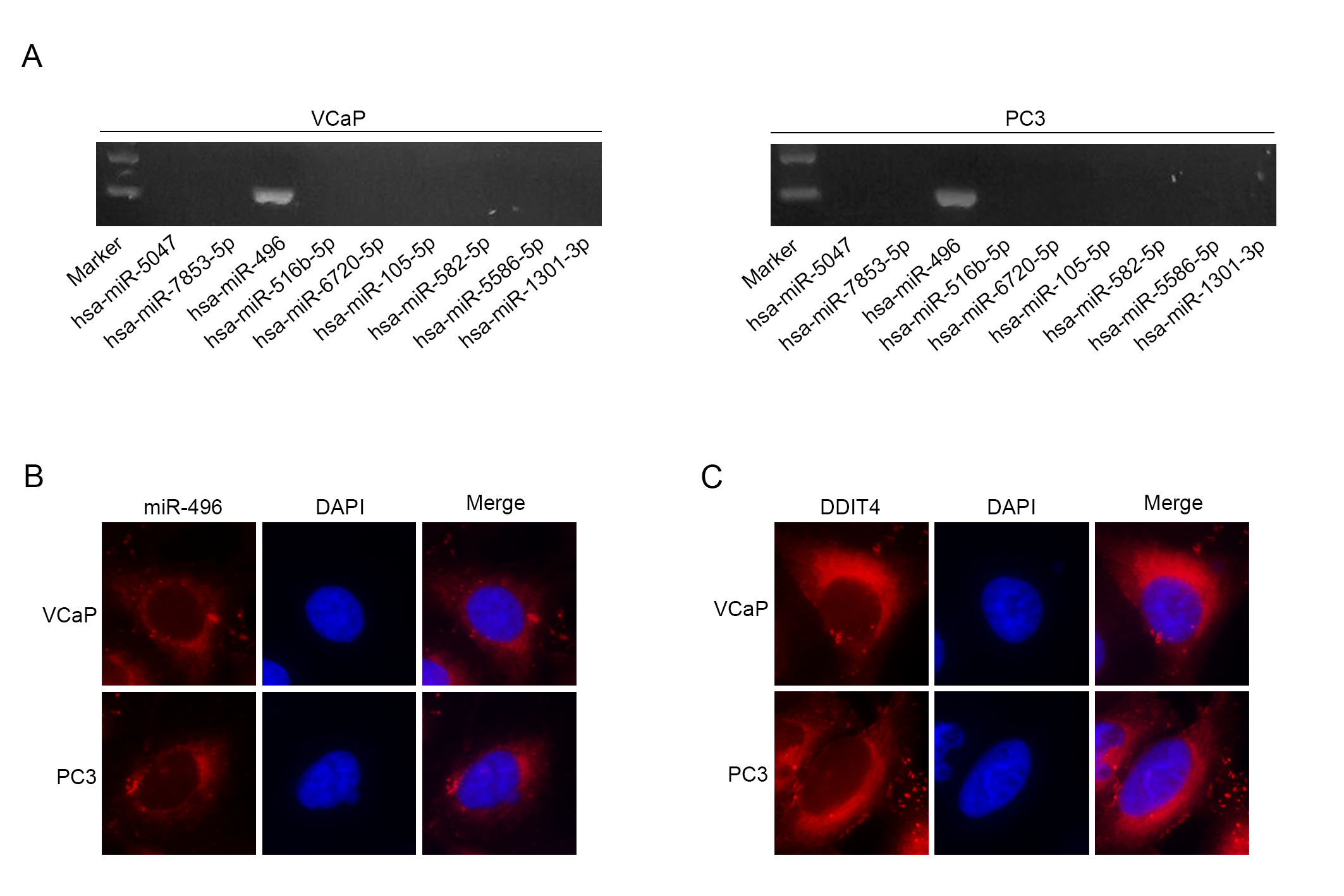

Supplement: Supplementary file 1 — Additional file 1 A. The blots of agarose gel electrophoresis for the evaluation of indicated miRNAs in the complexes from MS2-NNT-AS1 groups in MS2-RIP assays. B, C. FISH analyzed the cellular location of miR-496 and DDIT4 mRNA in VCaP and PC3 cells. [file 12935_2020_1505_MOESM1_ESM.tif]
